# Analysis of the Digital Footprint of Orthopaedic Surgeons

**DOI:** 10.5435/JAAOSGlobal-D-21-00063

**Published:** 2021-06-04

**Authors:** Ajith K. Subhash, Troy Sekimura, Trent M. Kajikawa, Rishi Trikha, Peter P. Hsiue, Amir Khoshbin, Christos Photopoulos, Alexandra Stavrakis

**Affiliations:** From the Department of Orthopaedic Surgery, David Geffen School of Medicine at the University of California Los Angeles, Los Angeles, CA (Mr. Subhash, Mr. Sekimura, Mr. Kajikawa, Dr. Trikha, Dr. Hsiue, and Dr. Stavrakis); the Division of Orthopaedic Surgery, University of Toronto, Toronto, ON, Canada (Dr. Khoshbin); and the Kerlan-Jobe Orthopaedic Institute, Los Angeles, CA (Dr. Photopoulos).

## Abstract

Patients increasingly rely on Google search to guide their choice of healthcare providers. Despite this trend, there is limited literature systematically characterizing the online presence of orthopaedic surgeons. The goal of this study was to identify the information patients see after queries of Google search when selecting orthopaedic surgeon providers.

**Methods:**

The Physician Comparable downloadable file from the Centers for Medicare and Medicaid Services was deduplicated and filtered. A list of orthopaedic surgeons within the United States was generated, of which a randomized sample was taken and queried using a Google Custom Search. The results for each surgeon's first page were classified into the following categories: (1) hospital-controlled content website, (2) third-party health website, (3) social media website, (4) primary academic journals, or (5) other.

**Results:**

The most frequently returned website was third-party health websites (43.3%). Statistically significant differences were observed in the categories across multiple comparisons, including academic and nonacademic orthopaedic surgeons, male and female providers, and surgeons from different graduation years.

**Discussion:**

Most of the results were attributed to third-party websites demonstrating that orthopaedic surgeons do not have notable control over their digital footprint. Increased patient visibility of physician-controlled websites and an objective rating system for patients remain potential areas of growth.

The internet has become vitally important in modern life. In 2020, an estimated 90% of US adults consistently used the internet, up from just 52% in 2000.^1^ People are increasingly using the internet to address health-related questions.^2^ It is estimated that up to 72% of internet users sought health-related information in 2013.^3^ Most of these queries were initiated on search engines, with the Google search engine being by far the most heavily used.^4^ Order of placement of search results has a notable effect on influence because 92% of users do not venture beyond the first page of search results.^5^

Previous studies examining the online presence of physicians in other specialties, such as radiology, radiation oncology, and neurosurgery, have demonstrated that physicians' online presence is often largely controlled by third-party websites, giving providers little control over their online identities. In addition, factors including practice setting and year of medical school graduation have been shown to affect the online footprint of medical professionals.^6,7^ For example, radiation oncologists who graduated in more recent years had an increasing proportion of social media websites among their top search results.^8^

The purpose of this study was to characterize the online presence of orthopaedic surgeons in the United States. We expect that the online presence of orthopaedic surgeons will be dominated largely by third-party sites that physicians themselves have little to no control over their online identities.

## Methods

The Physician Comparable downloadable file from the Centers for Medicare and Medicaid Services was accessed in January 2020. The Physician Comparable downloadable file captures approximately 90% of all physicians in the United States who are enrolled in Medicare fee for service.^9^ The file was deduplicated using providers' National Provider Identifier numbers and filtered across all specialty columns by “orthopaedic surgery.” From these criteria, a list of 23,640 orthopaedic surgeons within the United States was generated. Using G*Power 3.1.9.4, a power calculation (α = 0.05, df = 4, w = 0.1, and β = 0.1) was done, and accordingly a sample of 2,000 surgeons was randomly selected. Concatenation string search terms were constructed for each of these surgeons as follows: “first name” + “last name” + “credential” + “orthopaedic surgery” + “city” + “state” from the filtered list. Providers with no listed degree in the credential column were assigned “MD,” when concatenation terms were compiled, because most providers in the United States are MD licensed.^10^ These concatenation terms were then programmatically queried using a Google Custom Search Engine (Google) and Python 3.8.1. For each surgeon and their corresponding term, the first page of Google search results was extracted. Each surgeon's top 10 search results were then assigned into one of the following categories based on the domain name: (1) hospital, health, and physician-controlled content website; (2) third-party health and physician content website; (3) social media website; (4) primary academic journals; or (5) other (journal repositories, government websites, or other types) by two reviewers. If there was a discordance in the assignment, a third reviewer would examine the domain name for final classification.

A repository of orthopaedic surgeons with academic affiliations was also constructed. Programs were initially identified through a search of the Accreditation Council for Graduate Medical Education website. Subsequently, faculty were identified on individual department websites and confirmed with program directors and coordinators for accuracy. Only faculty with full-time appointments in their respective departments for orthopaedic surgery were included. This repository was then cross-compared with our sample of 2,000 surgeons by two reviewers to assign academic and nonacademic status to these providers.

A two-way χ^2^ analysis was done to determine any statistical difference between academic and nonacademic orthopaedic surgeons. Additional two-way χ^2^ analyses were done to determine any notable differences between male and female providers and also between graduation years from medical school for the providers. All analysis and data visualization were performed using R version 3.6.2 (RStudio).

## Results

The Centers for Medicare and Medicaid Services database revealed that 23,640 of the 1,141,140 physicians listed (2.07%) self-identified as orthopaedic surgeons. Of the 2,000 orthopaedic surgeons sampled from this population, 350 (17.50%) were classified as academic providers and 1650 (82.50%) were classified as nonacademic providers.

A total of 19,944 URLs were returned and categorized following the criteria listed in Supplemental Table 1 (http://links.lww.com/JG9/A142). For the 2,000 providers searched, 99.85% of them returned at least one Google result. Demographics for the sample are presented in Supplemental Table 2, http://links.lww.com/JG9/A142. The frequencies of the most common domains are presented in Supplemental Table 3, http://links.lww.com/JG9/A142. The most frequently returned domain was vitals.com constituting 11.1% of the total URLs. No primary academic journal websites were found in the top 10 most frequent domains.

**Figure 1:**
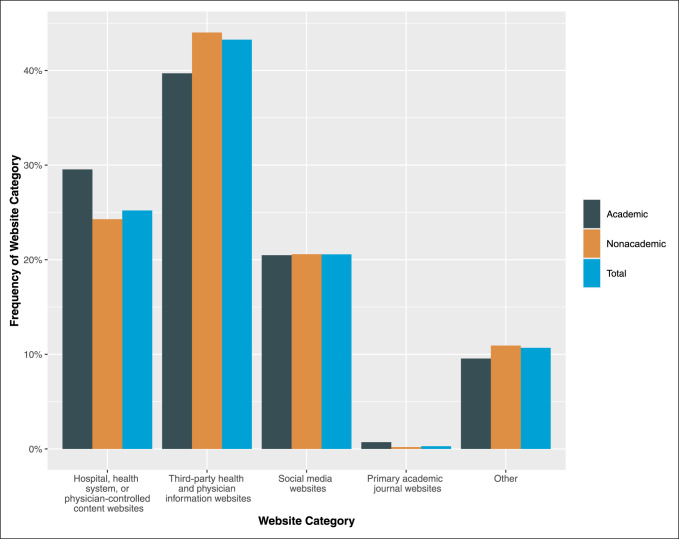
Graph showing the frequency of website category in top 10 search results for US orthopaedic surgeons.

Third-party physician and health websites were the most frequent search results and accounted for 8,628 of the results (43.3%). Hospital, health, and physician-controlled content websites accounted for 5,026 of the results (25.2%). Websites classified as social media accounted for 4,101 of the results (20.6%), and primary academic journals accounted for 57 results (0.3%). Websites belonging to the “other” category yielded 2,131 results (10.7%). These findings are listed in Figure 1.

A two-way χ^2^ analysis of the sample revealed a statistically significant difference in the categories between academic and nonacademic orthopaedic surgeons. One noticeable difference included hospital, health, or physician-controlled websites constituting 29.5% of the results for academic orthopaedic surgeons compared with 24.3% for nonacademic surgeons (*P* < 0.001) (Figure 1). In addition, a two-way χ^2^ analysis of the sample revealed statistically significant differences in the categories between male and female providers. A noticeable difference between these two cohorts included a higher frequency of third-party websites associated with male orthopaedic surgeons (43.6%) compared with 38.3% in female surgeons (*P* < 0.001). The last two-way χ^2^ analysis revealed statistically significant differences in the categories between the different graduation year sets found in Supplemental Table 2, http://links.lww.com/JG9/A142. Surgeons graduating between 2004 and 2018 had a much higher prevalence of hospital websites (29.0%) in their top 10 search results compared with surgeons graduating in earlier years (21.1% to 25.8%) (*P* < 0.001) (Figure 3).

**Figure 2:**
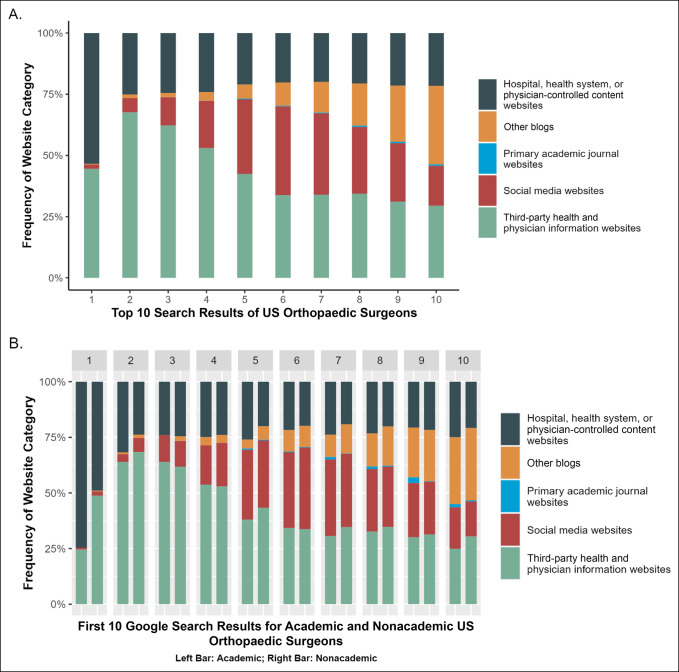
Graph showing **(A)** website categories by position within top 10 search results for all US orthopaedic surgeons and (**B**) website categories by position within top 10 search results for academic and nonacademic US orthopedic surgeons.

The most frequently returned URL in position 1 was classified as hospital, health, or physician-controlled content websites, whereas positions 2 to 5 most frequently returned a third-party health or physician information website. URLs in positions 7 to 9 also frequently returned a third-party health website. Only position 6 most frequently returned social media websites. All the top 10 search result frequencies by position for the sample of US orthopaedic surgeons are listed in Figure 2A.

Approximately 74.6% of first search results for academic orthopaedic surgeons were considered hospital, health, or physician-controlled websites compared with 48.9% for nonacademic surgeons. Third-party websites constituted 24.6% and 48.8% of first search results for academic and nonacademic surgeons, respectively. Social media websites in the first search result position were interestingly a small proportion for both academic and nonacademic surgeons at 0.8% and 1.9%, respectively. Of note, because the search position increases, the frequency of social media websites increases for all orthopaedic surgeons. These findings are summarized along with the top 10 search result frequencies by position and academic title status in Figure 2B.

**Figure 3:**
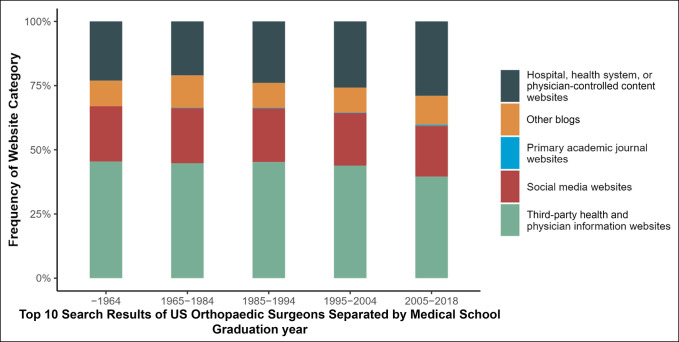
Graph showing the frequency of website category in top 10 search results for US orthopaedic surgeons by medical school graduation year.

For surgeons who graduated from 1964 to 2018, the most prevalent websites were third-party health and physician websites. In addition, across all graduation years, the frequency of social media websites was similar ranging from 19.7% to 21.5%. These findings along with the top 10 search result frequencies for the sample of orthopaedic surgeons categorized by medical school graduation year can be found in Figure 3.

## Discussion

To the best of our knowledge, this is the first study to characterize the online presence of US orthopaedic surgeons. These findings are of particular importance given that we are in an increasingly digital society, where patients are more likely to use the internet to seek health information.^11^ Analysis revealed that most orthopaedic surgeons lacked notable control over their online identity, as evidenced by their most frequently returned website type being a third-party health or physician information website. This applied to both academic (39.7% of total links) and nonacademic (44.0% of total links) orthopaedic surgeons within the United States. These results are consistent with studies conducted by Prabhu et al, Vijayasarathi et al, and Kim et al, who examined the online presence of radiation oncologists, radiologists, and neurosurgeons, respectively. In contrast to these other specialties, however, orthopaedic surgeons seemed to exert more control on their first page in the form of social media. Social media websites comprised a total of 20.6% of the links returned and had an approximately similar frequency for both academic and nonacademic orthopaedic surgeons in the United States.

Physicians and hospitals could ensure that each page corresponding to a specific provider has the most relevant and updated information relating to them. However, this is not always the case.

Another means for surgeons to influence the type of information patients see is through social media websites. There are understandable concerns regarding the physician's usage of social media. Opponents criticize that physicians use social media because of the risk of compromising patient confidentiality, privacy, and medicolegal challenges.^12,13^ However, studies have shown that social media is particularly underutilized in orthopaedics despite its massive opportunity to play an important role in medical education, rapport building, and helping patients and their families to navigate orthopaedic conditions.^14-16^

In addition, previous studies have highlighted the problematic reliance on third-party websites. For example, some “reviews” on third-party websites are purchased artificial positive reviews, or on the other hand, from competitors or other saboteurs in an attempt to lower other physician's credibility.^17,18^ Others have noted that some physician-rating websites are hosted by insurance companies, which raises questions regarding conflicts of interest because insurance companies may be motivated to drive patients to cheap providers rather than seeking quality care.^19^ Finally, websites such as zocdoc.com, one of our most frequently returned website results, often times require physicians to pay the website to be listed on their site and to allow patients to directly schedule appointments through their site.^20^ There have been reports that zocdoc.com, for example, has a bias toward positive reviews for a multitude of reasons. One explanation is that, due to the financial incentive, physician profiles may be artificially inflated.^21^ Therefore, patients should be wary while using physician search platforms such as this.

With the many drawbacks of third-party rating websites highlighted, there is a need for a more objective rating system of orthopaedic surgeons for patients. This objective rating system may include statistics on overall complication rates, surgical complication rates, volume of specific procedures, and real objective patient-reported outcomes, in addition to the traditional patient satisfaction ratings provided for each provider. Furthermore, with the advent of search engine optimization, surgeons could potentially modify their online presence so that patients are able to see a higher prevalence of physician-controlled websites first. This may be one tool that orthopaedic surgeons can use to improve patient-provider communication and build rapport with patients.

There are several limitations associated with this study. First, as in other studies, the study population has a selection bias because the list of orthopaedic surgeons' constructed relies on self-reporting.^9^ In addition, the concatenation string search terms that were constructed may not exactly capture how patients would seek health information on the website. Furthermore, the Google Custom Search Engine is the only interface that allows users to programmatically query Google at a large scale that abides to their terms of service. As such, using their Custom Search Engine returns slightly different results as noted by their documentation compared with a traditional Google search through their main search engine. Another limitation with Google search is that previous searches and a user's geographical parameters may alter the results returned to create a personalized list for the user. This study did not factor for these modifications during our queries.

Future directions include a future reevaluation of the online presence of orthopaedic surgeons because of the continued massive influx of health information online and ever-changing nature of the internet. In addition, the online presence of orthopaedic surgeons in the United States could be compared with those worldwide.

Understanding the distribution of Google search results is critical to appreciating the online presence of orthopaedic surgeons. Increased patient visibility of physician-controlled websites, which provide a platform for valuable tools, such as patient education and improving patient-provider communication, and a robust objective rating system for patients remain potential areas of growth.

## Summary

Orthopaedic surgeons do not have notable control over their online presence, and there is a need for an objective rating system for patients to reliably use.

## Supplementary Material

SUPPLEMENTARY MATERIAL
