# Supplementary material for: Analysis of the Digital Footprint of Orthopaedic Surgeons
Source: J Am Acad Orthop Surg Glob Res Rev. 2021 Jun 4;5(6):e21.00063. doi: 10.5435/JAAOSGlobal-D-21-00063 (PMC8183712; doi:10.5435/JAAOSGlobal-D-21-00063)
Supplement: SUPPLEMENTARY MATERIAL [file jagrr-5-e21.00063-s001.docx]

| **Table 1.** | Website categories | |  |  |  |
| --- | --- | --- | --- | --- | --- |
| Type |  | Category |  |  | Examples |
| 1 |  | hospital, health, physician-controlled content website |  |  | Hospital, health-care network, university, and physician sites (hss.edu,  mayoclinic.com, uclahealth.org) |
| 2 |  | third-party health and physician content website |  |  | vitals.com, healthgrades.com, health.usnews.com, medicinenet.com |
| 3 |  | social media website |  |  | sharecare.com, doximity.com, facebook.com, linkedin.com, youtube.com |
| 4 |  | primary academic journals |  |  | arthroplastyjournal.org, ijssurgery.com |
| 5 |  | Other |  |  | yellowpages.com, journal repositories, government websites, blogs,  meeting programs |

**Table 1.** Website categories with corresponding examples for each domain name extracted

| **Table 2.** | Demographic characteristics of U.S. orthopaedic surgeons | | | | |
| --- | --- | --- | --- | --- | --- |
|  | Characteristic |  |  |  | Value (%) |
| Total number of U.S. orthopaedic surgeons | | | |  | 23,640 |
| Sample |  |  |  |  | 2000 (100%) |
| Sex |  |  |  |  |  |
|  | Male |  |  |  | 1859 (92.95%) |
|  | Female |  |  |  | 141 (7.05%) |
| Degree type |  |  |  |  |  |
|  | MD |  |  |  | 673 (33.65%) |
|  | DO |  |  |  | 33 (1.65%) |
|  | Other |  |  |  | 1 (0.05%) |
|  | None listed | |  |  | 1293 (64.65%) |
| Academic affiliation | |  |  |  |  |
|  | Academic | |  |  | 350 (17.50% |
|  | Nonacademic | |  |  | 1650 (82.50%) |
| Graduation year from medical school | | | |  |  |
|  | Before 1964 | |  |  | 13 (0.65%) |
|  | 1964-1983 | |  |  | 403 (20.15%) |
|  | 1984-1993 | |  |  | 485 (24.25%) |
|  | 1994-2003 | |  |  | 564 (28.20%) |
|  | 2004-2017 | |  |  | 527 (26.35%) |
|  | Graduation year not listed | | |  | 8 (0.40%) |
| Google search results retrieved | | |  |  |  |
|  | 0 |  |  |  | 3 (0.15%) |
|  | 1 |  |  |  | 1 (0.05%) |
|  | 2 |  |  |  | 0 (0.00%) |
|  | 3 |  |  |  | 0 (0.00%) |
|  | 4 |  |  |  | 0 (0.00%) |
|  | 5 |  |  |  | 0 (0.00%) |
|  | 6 |  |  |  | 2 (0.10%) |
|  | 7 |  |  |  | 0 (0.00%) |
|  | 8 |  |  |  | 3 (0.15%) |
|  | 9 |  |  |  | 3 (0.15%) |
|  | 10 |  |  |  | 1988 (99.40%) |

| **Table 3.** | Top 10 domains for the first page of Google search results for U.S. orthopaedic surgeons | | | |
| --- | --- | --- | --- | --- |
| Rank | Domain name | Category | Number of hits | Number of orthopaedic surgeons (n=2000) |
| 1 | [www.vitals.com](http://www.vitals.com/) | 2 | 2214 | 1745 (87.38) |
| 2 | [www.sharecare.com](http://www.sharecare.com/) | 3 | 1982 | 1448 (72.51) |
| 3 | [www.healthgrades.com](http://www.healthgrades.com/) | 2 | 1967 | 1734 (86.83) |
| 4 | [health.usnews.com](http://health.usnews.com/) | 2 | 1689 | 1568 (78.52) |
| 5 | [www.doximity.com](http://www.doximity.com/) | 3 | 1600 | 1555 (77.87) |
| 6 | [www.medicinenet.com](http://www.medicinenet.com/) | 2 | 647 | 587 (29.39) |
| 7 | [doctor.webmd.com](http://doctor.webmd.com/) | 2 | 535 | 444 (22.23) |
| 8 | [www.yellowpages.com](http://www.yellowpages.com/) | 5 | 435 | 376 (18.83) |
| 9 | [www.zocdoc.com](http://www.zocdoc.com/) | 2 | 358 | 344 (17.23) |
| 10 | [www.caredash.com](http://www.caredash.com/) | 2 | 249 | 239 (11.97) |
